# Evaluation of Camptothecin Through Computational and Experimental Approaches Targeting Membrane Receptors on Breast Cancer Cells for Potential Therapeutic Applications

**DOI:** 10.3390/ijms27177857

**Published:** 2026-09-02

**Authors:** Elmer Joel Millan-Casarrubias, Lucero Ruiz-Mazón, Eduardo Pérez Salazar, Pedro Cortés Reynosa, Yazmín Mariela Hernández-Rodríguez, Oscar Eduardo Cigarroa-Mayorga

**Affiliations:** 1 Department of Advanced Technologies, UPIITA—Instituto Politécnico Nacional, Av. IPN 2580, Mexico City 07340, Mexico; emillan@cinvestav.mx (E.J.M.-C.); yazmin.hernandez@cinvestav.mx (Y.M.H.-R.); 2 Departamento de Biología Celular, Centro de Investigación y de Estudios Avanzados del Instituto Politécnico Nacional (CINVESTAV-IPN), Mexico City 07360, Mexico; lucero.ruiz@cinvestav.mx (L.R.-M.); eduardo.perez@cinvestav.mx (E.P.S.); pedro.cortes@cinvestav.mx (P.C.R.)

**Keywords:** camptothecin, molecular docking, EGFR, HER-2, integrin, estrogen receptor, breast cancer

## Abstract

Breast cancer remains among the leading causes of incidence and mortality worldwide. Consequently, identifying new treatments and strategies is of critical importance. Evidence indicates that camptothecin and its derivatives may exert anticancer effects in various cancer cell lines, including colon, lung, and ovarian cancers. However, their effects in breast cancer are not yet fully understood. Prior theoretical studies employing docking and molecular dynamics suggest that camptothecin could bind to the HER2 and EGFR receptors, which are overexpressed in breast cancer cells. Investigating interactions between novel molecules with affinity for membrane receptors overexpressed in breast cancer is important for developing personalized therapies and for advancing strategies to selectively target nanomaterials to these cells for diagnostic and therapeutic purposes. This study evaluated the in silico and in vitro effects of camptothecin on the MCF-7 and MDA-MB-231 breast cancer cell lines. Our results show significant inhibition of proliferation and reduced migration at 24, 48, and 72 h in both cell lines. The theoretical analysis indicates high affinity of camptothecin for receptors overexpressed in breast cancer compared with current treatments.

## 1. Introduction

Camptothecin is a naturally occurring alkaloid derived from Camptotheca acuminata (also known as Camptotheca or Queen’s Crepe Myrtle), native to China [1]. This Nyssaceae plant has been used in traditional Chinese medicine for centuries. Isolated in the 1960s, camptothecin exhibits potent anticancer activity by inhibiting topoisomerase I [2], a key enzyme in DNA replication [3,4,5,6]. Its initial clinical use was limited by marked toxicity. Camptothecin and its derivatives play important roles in medicine as anticancer agents; among the most widely used derivatives are irinotecan and topotecan, which are employed against colorectal, lung, ovarian, and cervical cancers [4,7,8,9]. Ongoing research aims to improve efficacy, reduce toxicity, and explore new formulations and combination therapies, as well as potential applications in other diseases characterized by uncontrolled cell proliferation [10,11]. In this context, breast cancer is a highly prevalent cancer and a leading cause of cancer-related mortality among women worldwide. According to the World Health Organization (WHO), breast cancer affects millions of women and, to a lesser extent, men, with rising incidence in many regions [12]. Risk factors include family history, hormonal changes, lifestyle, and exposure to certain carcinogens [12]. Early detection through mammography and awareness of warning signs are crucial for improving survival rates [13]. Despite advances in diagnosis and treatment increasing cure rates in many countries, disparities in access to healthcare remain a global challenge. In breast cancer, the principal receptors expressed on tumor cells are the estrogen receptor (ER), progesterone receptor (PR), and HER2 [14,15,16]. The expression of these receptors is essential for classifying breast cancer, predicting prognosis, and guiding treatment. ER and PR-positive tumors typically respond to hormonal therapies that block hormonal signaling, while HER2-overexpressing tumors are treated with targeted therapies such as trastuzumab [17,18,19]. Assessing receptor status is crucial for personalizing treatment and improving outcomes. Receptor-targeted therapies have transformed breast cancer care. For ER/PR-positive tumors, hormonal agents like tamoxifen, aromatase inhibitors, and fulvestrant inhibit the signaling that promotes tumor growth [20,21]. For HER2-positive cancers, therapies such as trastuzumab, pertuzumab, and other monoclonal antibodies or tyrosine kinase inhibitors block HER2 activity and reduce proliferation [22,23,24]. Personalized therapies have substantially improved survival and quality of life for breast cancer patients, offering more effective treatments with fewer side effects than traditional approaches. Ongoing research and development of inhibitors targeting receptors overexpressed in breast cancer (ER, PR, and HER2) remain essential. Tumor heterogeneity and acquired resistance can limit current therapies, underscoring the need for new inhibitors and targeted strategies to enhance personalization, overcome resistance mechanisms, reduce adverse effects, and increase response rates and survival. These advances also broaden therapeutic options for patients with advanced disease or those who do not respond to existing treatments, strengthening efforts to combat breast cancer and improve patient outcomes. In this study, we assessed the cytotoxic effects of camptothecin on the MDA-MB-231 and MCF-7 breast cancer cell lines. A wound-healing assay was also performed to evaluate camptothecin’s impact on cell migration at 24 h. Additionally, an in silico analysis was conducted to compare camptothecin’s affinity for membrane receptors expressed in breast cancer cells EGFR, ER, HER2, and integrins against those of established anticancer drugs.

## 2. Results

### 2.1. Molecular Docking

#### 2.1.1. Coupling Energies (ΔG) and Dissociation Constants of Ligands Against the HER2 and EGFR Receptors

Table 1 summarizes the docking results for the complete structures of the HER2 and EGFR receptors, including binding energies and dissociation constants (Kd) for the breast-cancer drugs tested and for camptothecin. For HER2, camptothecin exhibited the most favorable binding energy, while erlotinib showed the lowest predicted affinity, with TAK in proximity. For EGFR, camptothecin again demonstrated the highest affinity among the tested compounds, whereas TAK showed the lowest predicted affinity.

Table 2 presents the binding energies and affinity constants (Kd) for the drugs tested against ER and integrin receptors. Camptothecin exhibited the strongest binding to integrins among the ligands. For the estrogen receptor, analyses were performed on two structures, G2 and G7. In the G2-ER, camptothecin had the most favorable binding energy, with neratinib closely approaching it, while TAK showed the least favorable energy. In the G7-ER, neratinib yielded the best binding energy, followed by TAK, with erlotinib showing the weakest affinity. Across receptors, camptothecin generally showed the most favorable binding energy, notably for EGFR and then HER2, relative to other targets. Overall, although each drug has its distinct mechanism of action in treating breast cancer, camptothecin demonstrated comparatively higher affinity and more favorable binding energies than the other compounds, suggesting potential receptor interactions or cellular uptake pathways in breast cancer cells.

#### 2.1.2. Molecular Interactions Between Camptothecin and HER2

**Figure 1:**
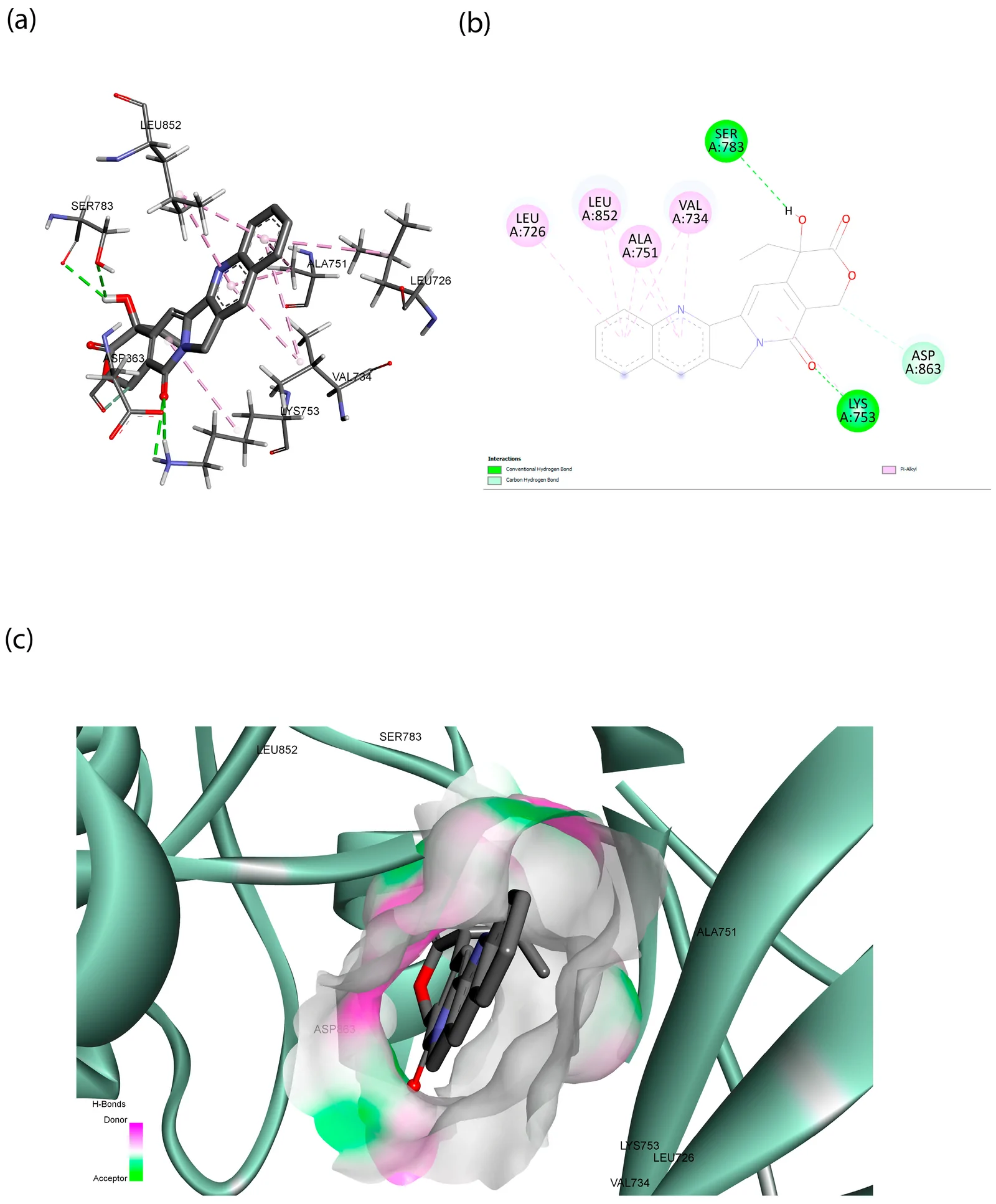
2D interaction diagram between camptothecin and the HER2 receptor. Hydrogen bond, van der Waals, pi-alkyl, and pi-sigma were the interactions obtained between camptothecin and the HER2 receptor.

Figure 1 presents a spatial view Figure 1a showing camptothecin anchored in the receptor cavity. The ligand backbone is oriented to allow functional groups to contact surrounding polar and nonpolar residues. The bipartite regions and aromatic rings are oriented toward the hydrophobic portion of the cavity, indicating good geometric fit and volume complementarity. Figure 1b reveals hydrogen bonds between the ligand and residues SER 783 and LYS 753 (highlighted in green), suggesting hydrogen-bond donor/acceptor interactions that contribute to the ligand’s specificity and orientation within the binding site. The presence of SER 783 and LYS 753 indicates an additional polar layer at the cavity periphery, which may promote interaction stability. The pink-colored amino acids denote residues that form π-alkyl interactions between the ligand’s aromatic rings and nearby hydrophobic residues, primarily LEU 726, LEU 852, ALA 751, and VAL 734. These hydrophobic contacts help lock the ligand’s orientation and enhance overall stability by complementing the cavity geometry.

Figure 1c provides a three-dimensional view of the cavity surrounding the ligand and the associated charge/color distribution, which highlights regions of potential electron density delocalization or areas of stronger interaction. The pink region denotes the proton-donating portion of the protein, the green region indicates the proton-accepting portion, and the white surface marks the hydrophobic or nonpolar portion of the HER2 protein. The ligand sits within a mixed hydrophobic/hydrophilic environment, with coordinated interactions around the ligand’s central region that promote a well-defined orientation within the cavity.

#### 2.1.3. Molecular Interactions Between Camptothecin and EGFR

**Figure 2:**
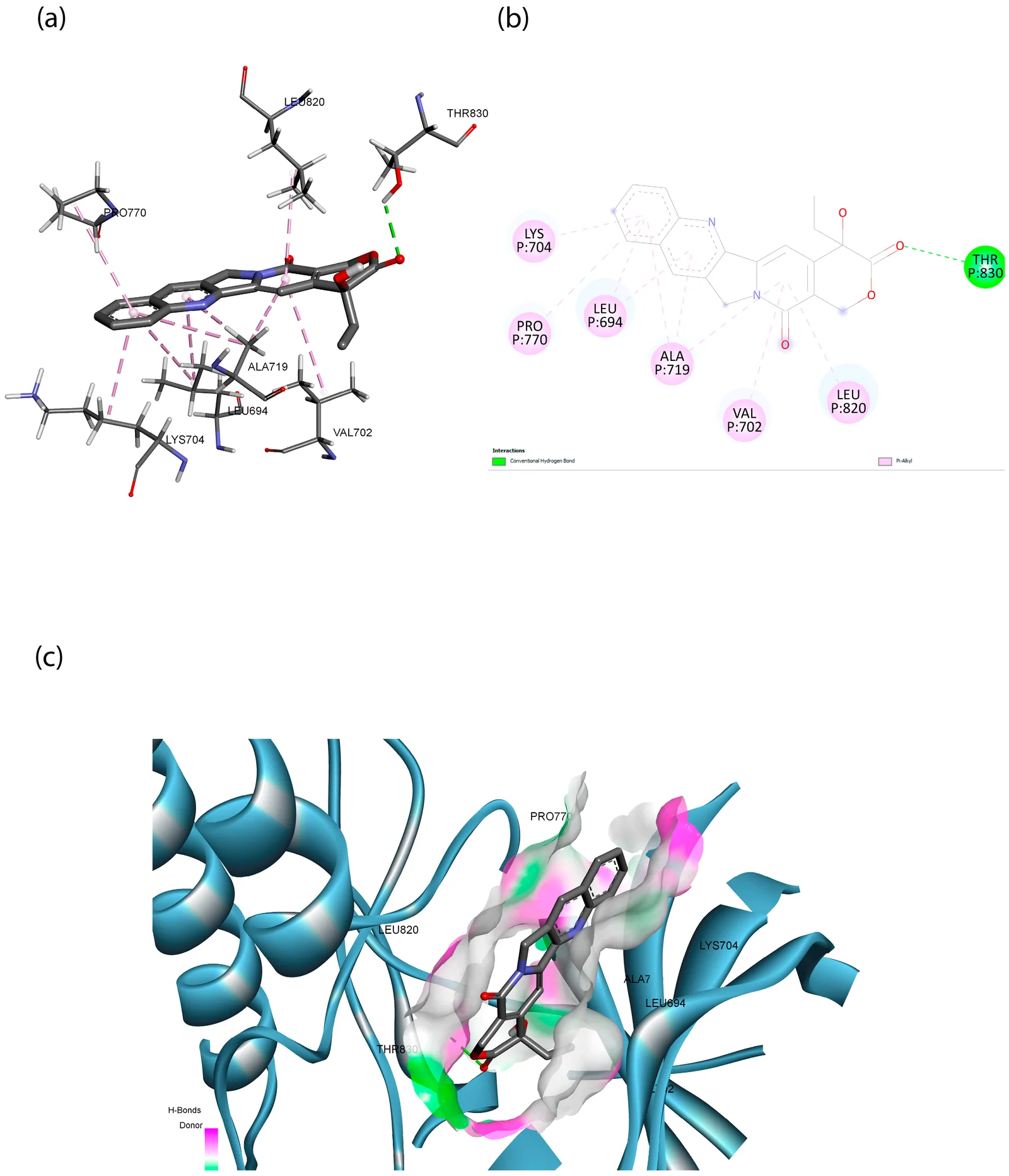
(**a**) Three-dimensional representation of camptothecin positioned within the predicted EGFR binding pocket. (**b**) Two-dimensional interaction map illustrating a conventional hydrogen bond with Thr830 and predominantly hydrophobic π–alkyl interactions involving residues such as Lys704, Leu694, Pro770, Ala719, Val702, and Leu820. (**c**) Three-dimensional surface representation of the binding pocket showing the spatial accommodation of camptothecin within EGFR.

Figure 2 shows that camptothecin anchors within the EGFR binding pocket via pi-alkyl contacts with several hydrophobic residues, including Lys 704, Leu 694, Pro 770, Ala 719, Val 702, and Leu 820. These interactions are depicted across multiple typographic representations to emphasize the spatial arrangement of the aromatic rings relative to these residues (Figure 2a,b). A single hydrogen bond is formed with Thr 830, contributing to binding specificity. Figure 2c provides a surface representation in which the proton-donating region of the protein is colored pink, the proton-accepting region green, and the hydrophobic region white, suggesting that the proton network at the interface may influence ligand docking and complex stability. Overall, camptothecin binding to EGFR is supported by a network of nonpolar pi-alkyl contacts with surrounding hydrophobic residues, complemented by a hydrogen-bonding interaction that imparts specificity, and by a distribution of proton donors/acceptors at the interface that could modulate affinity. These observations advance the structural understanding of ligand–receptor recognition and may inform optimization strategies for anti-EGFR ligands.

#### 2.1.4. Molecular Interactions Between Camptothecin and Estrogen Receptor 2

**Figure 3:**
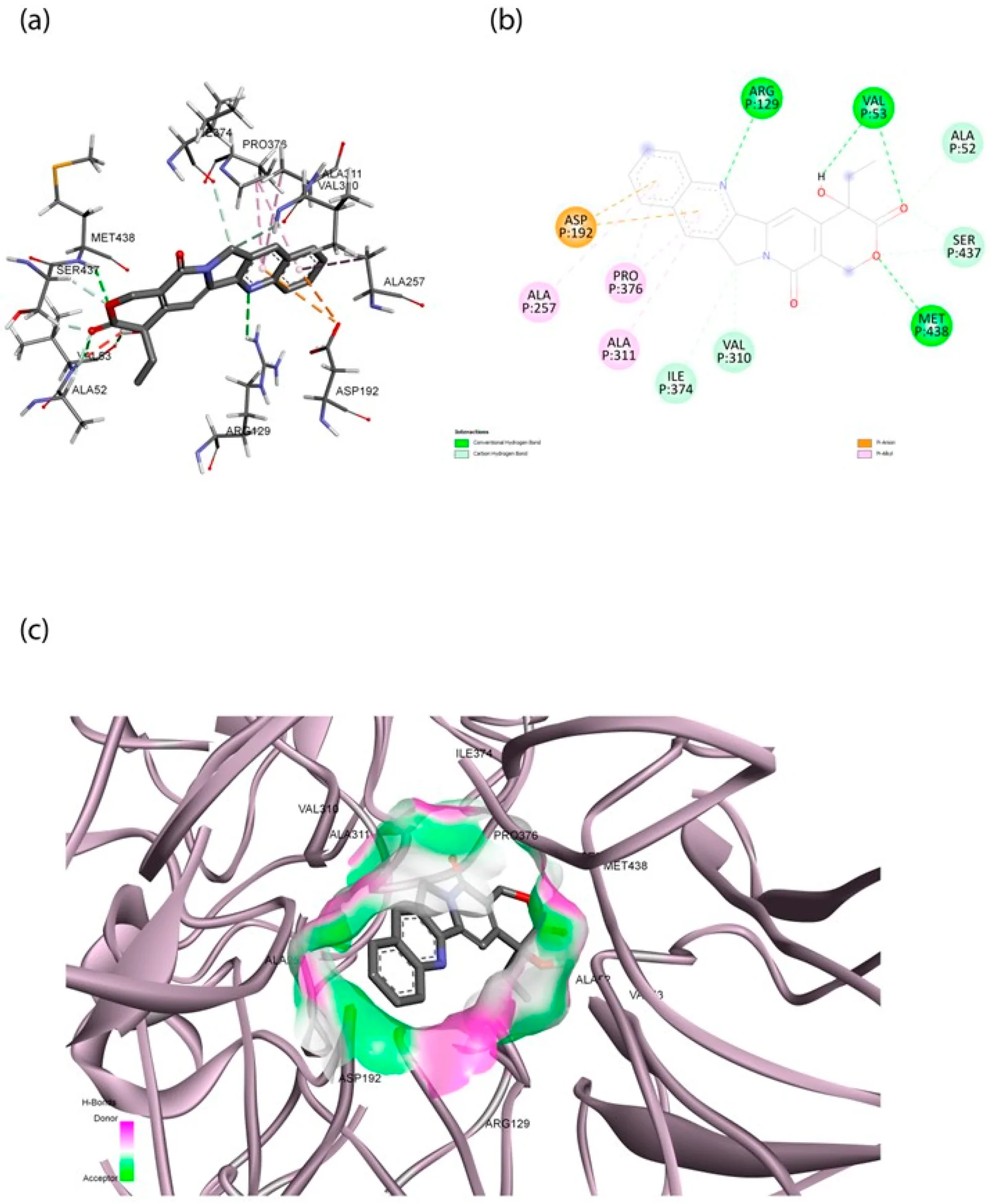
(**a**) Three-dimensional representation of camptothecin within the predicted binding pocket. (**b**) Two-dimensional interaction map highlighting hydrogen bonds, van der Waals contacts, and π-related interactions between camptothecin and residues within the binding site. (**c**) Three-dimensional surface representation of the binding pocket illustrating the spatial accommodation of camptothecin and the distribution of hydrogen-bond donor and acceptor regions around the ligand.

Figure 3 illustrates a three-dimensional representation of the camptothecin–estrogen receptor 2 complex. Figure 3a depicts the complex formed between camptothecin and the ligand-binding domain of estrogen receptor 2, illustrating the three-dimensional orientation of the ligand within the receptor’s active-site cavity and the positioning of key camptothecin functional groups that facilitate specific interactions with the binding pocket. Figure 3b presents a two-dimensional diagram of the binding residues, highlighting interactions between camptothecin and residues of the overexpressed ERβ receptor. Notable interactions include pi-alkyl contacts with Leu 391, Met 388, Leu 387, and Ala 350. These aromatic or ring-based interactions with aliphatic moieties stabilize the camptothecin–ERβ complex within the hydrophobic region of the active pocket. Pi–sulfur contacts are observed with Cys 530, and the Cys side chain may contribute to dispersion energies and to potential pi–sulfur interactions. Hydrogen bonds involve Gly 521 as part of a hydrogen-bond stabilization network linking camptothecin functional groups with those of the receptor. Figure 3c presents a conformational/channeling representation of the interaction, offering an enveloping helical/serpentine view of the ERβ domain with camptothecin accommodated in its pocket. The key residues—Leu 391, Met 388, Leu 387, Ala 350, Cys 530, and Gly 521—are clearly identified, and the hydrogen-bond network contributing to complex stability is depicted. The orientation of camptothecin relative to the receptor’s helical axis and the complementary nature of the pi–alkyl and pi–sulfur interactions with the stereochemistry of the coupling are evident.

#### 2.1.5. Molecular Interactions Between Camptothecin and Estrogen Receptor 7

**Figure 4:**
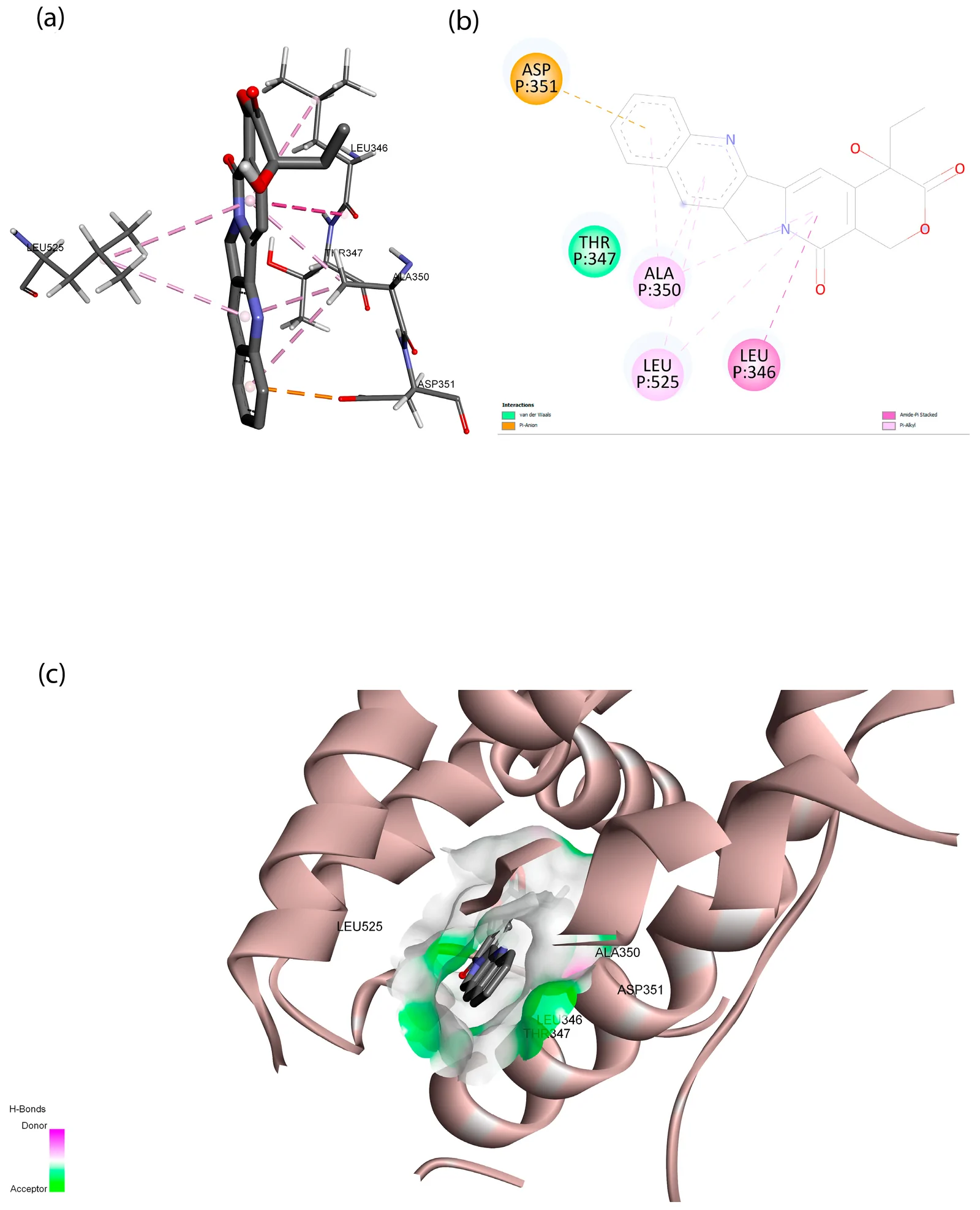
2D interaction diagram between camptothecin and the Estrogen Receptor 7. Hydrogen bond, van der Waals, pi-alkyl, and pi-sigma were the interactions obtained between camptothecin and the Estrogen Receptor 7 receptor.

Figure 4 depicts the spectrum of interactions between camptothecin, and Estrogen Receptor 7 (ER7) as revealed by a detailed 2D interaction map. The figure catalogs the specific amino acid residues that contact camptothecin and classifies the interaction types observed. Figure 4a presents a three-dimensional view of camptothecin within the ER7 binding site, with stabilization largely attributable to hydrophobic contacts involving camptothecin’s aromatic moieties. Figure 4b provides a two-dimensional interaction map showing contacts between camptothecin and ER7 residues. The predominant interactions are hydrophobic, including pi–alkyl contacts with ALA 350, LEU 525, and LEU 346, as well as a pi–anion interaction with ASP 351. Figure 4c shows the protein surface, highlighting the interaction network within the binding pocket and the residues in proximity to camptothecin. Regions responsible for these interactions are accentuated to illustrate the contact topology. Overall, the complex is characterized by predominantly hydrophobic interactions.

#### 2.1.6. Molecular Interactions Between Camptothecin and Integrin

**Figure 5:**
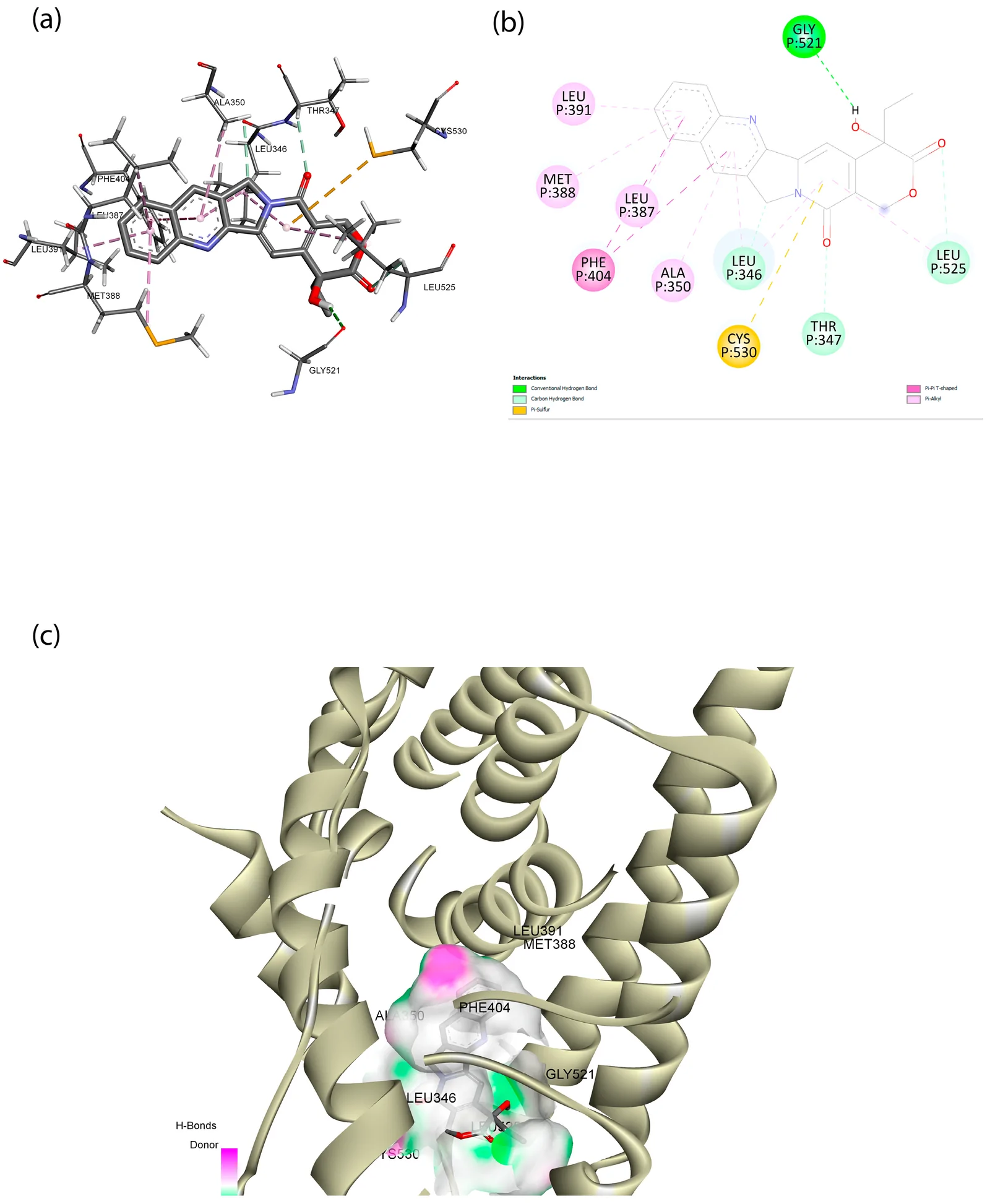
2D interaction diagram between camptothecin and the Integrin receptor. Hydrogen bond, van der Waals, pi-alkyl, and pi-sigma were the interactions obtained between camptothecin and the Integrin receptor.

Figure 5 depicts the complex formed between camptothecin and integrin, illustrating a crucial step toward understanding how this ligand may modulate adhesion and signaling pathways implicated in breast cancer progression. Camptothecin is situated within a hydrophobic pocket of the protein, indicating a combination of hydrophobic interactions and stabilization by hydrogen bonds. Figure 5 presents a three-panel model (a–c) of the binding between the camptothecin molecule and the active site of integrin αvβ3 within a breast cancer-relevant context. Figure 5a shows a three-dimensional representation of camptothecin at the αvβ3 binding site, illustrating the ligand’s overlap with the cavity and the surrounding envelope of proximal amino acid residues. Figure 5b provides a two-dimensional interaction map detailing contacts between camptothecin and protein residues. Highlighted interactions include pi-alkyl, pi-anion, and hydrogen bonds with catalytic or structural residues. Figure 5c displays the protein surface, emphasizing the interaction network within the binding pocket where camptothecin is encapsulated by nearby residues; the regions implicated in the interactions are highlighted. The pink region of the integrin corresponds to the proton-donor surface, while the green region denotes the proton-acceptor surface relative to the ligand.

#### 2.1.7. Molecular Dynamics Results for Protein–Camptothecin Complexes

**Figure 6:**
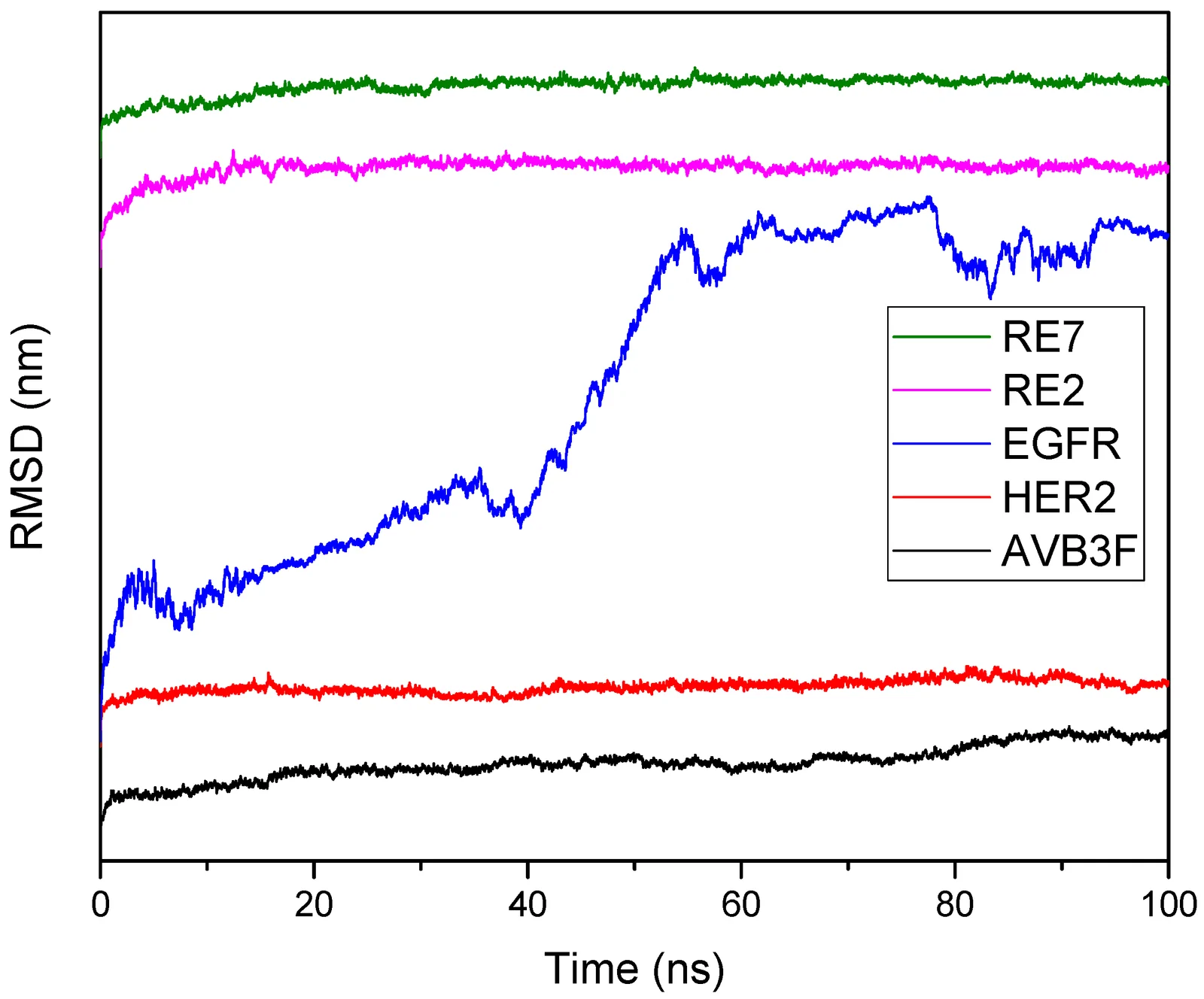
RMSD analysis of camptothecin complexes with membrane receptors. The plot summarizes the stability of the complexes formed during molecular modeling, as indicated by RMSD over time. The green trajectory corresponds to the CPT–estrogen receptor 7 complex, the pink trajectory to the CPT–estrogen receptor 2 complex, the blue trajectory to the CPT–EGFR complex, the red trajectory to the CPT–HER2 complex, and the black trajectory to the CPT–AVB3F integrin receptor complex.

Figure 6 presents the results from molecular dynamics simulations of the complexes formed after molecular modeling. The representative conformation selected for each complex corresponds to the structure with the most favorable binding energy. Root-mean-square deviation (RMSD) analysis quantifies the extent of structural deviation of the protein–ligand complex in nanometers (nm). In this context, an RMSD exceeding 2 Å would indicate reduced stability and potential dissociation of the complex over time. The observed RMSD values for all complexes remained below 2 Å, indicating theoretical stability, with the exception of the CPT–EGFR complex, which exhibited comparatively higher fluctuations. Notably, although this complex demonstrated the greatest variability, the RMSD did not exceed 2 Å.

#### 2.1.8. Viability Assays in the MCF-7 Breast Cancer Cell Line

**Figure 7:**
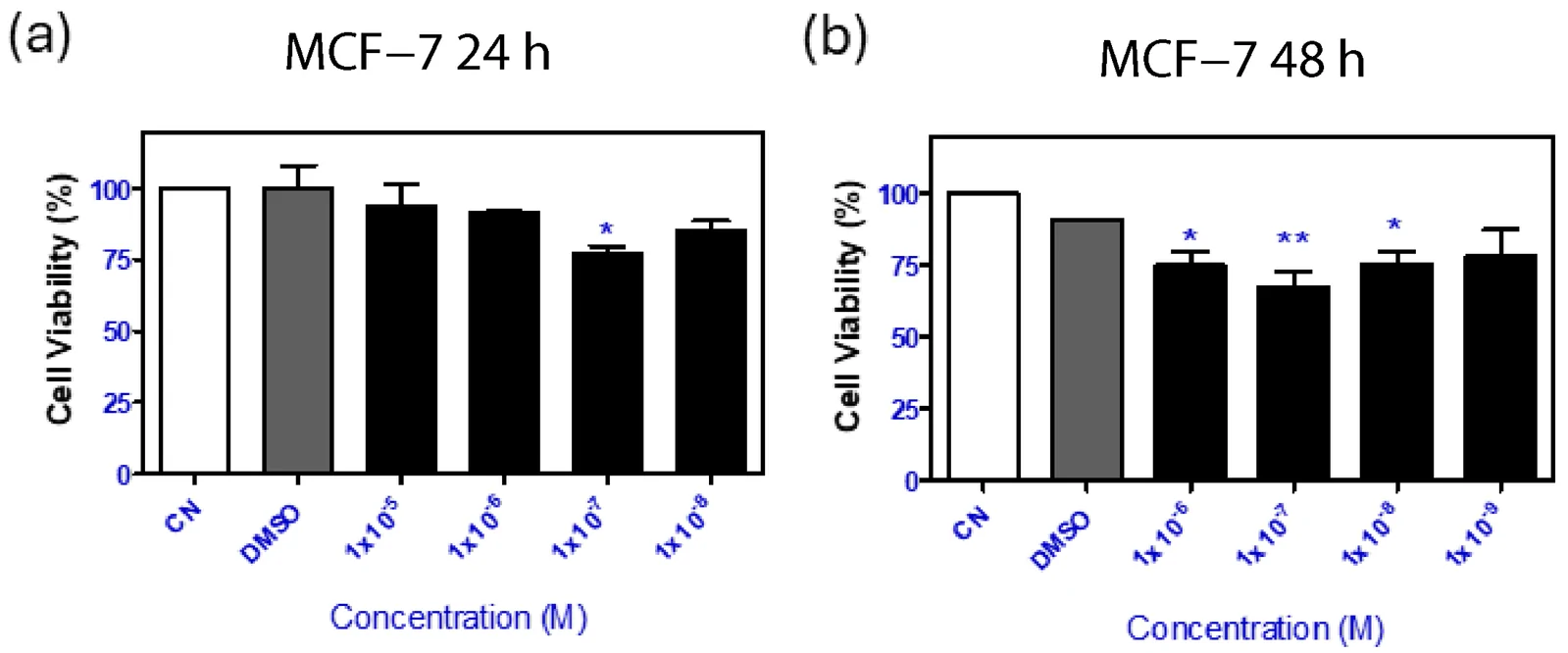
24 and 48 h cell viability of MCF-7 breast cancer cells treated with camptothecin, assessed by MTT assay. Figure (**a**) shows viability at 24 h post-treatment with camptothecin dissolved in DMSO. Figure (**b**) shows viability at 48 h post-treatment. Assays were performed in triplicate, and statistical analysis included ANOVA followed by Tukey’s post hoc test at a 95% confidence level (* *p* < 0.05) (** *p* < 0.01).

Figure 7 presents the viability assay results at 24 h (Figure 7a) and 48 h (Figure 7b) in the MCF-7 cell line following exposure to camptothecin at concentrations ranging from 1 × 10^−8^ to 1 × 10^−5^ M. Upon reaching approximately 90% confluence, cells were serum-starved 12 h before treatment by reducing fetal bovine serum concentration. Camptothecin was dissolved in DMSO as the vehicle. Figure 7a (24 h time point) shows no statistically significant effect across concentrations, with a notable exception at 1 × 10^−7^ M, where an inhibition of about 25% was observed. Figure 7b (48 h time point) reveals a statistically significant, concentration-dependent reduction in viability, with the greatest growth inhibition at 1 × 10^−7^ M, yielding roughly 30% inhibition relative to control. Overall, the effect was concentration-dependent but did not exceed 30%, rendering the cell line unsuitable for further study. It is noted that the assays were performed in triplicate. The inhibitory effect observed in this cell line was relatively modest, with an estimated IC_50_ value of 4.18 × 10^−4^ M.

#### 2.1.9. Viability Assays in MDA-MB-231 Breast Cancer Cell Lines

**Figure 8:**
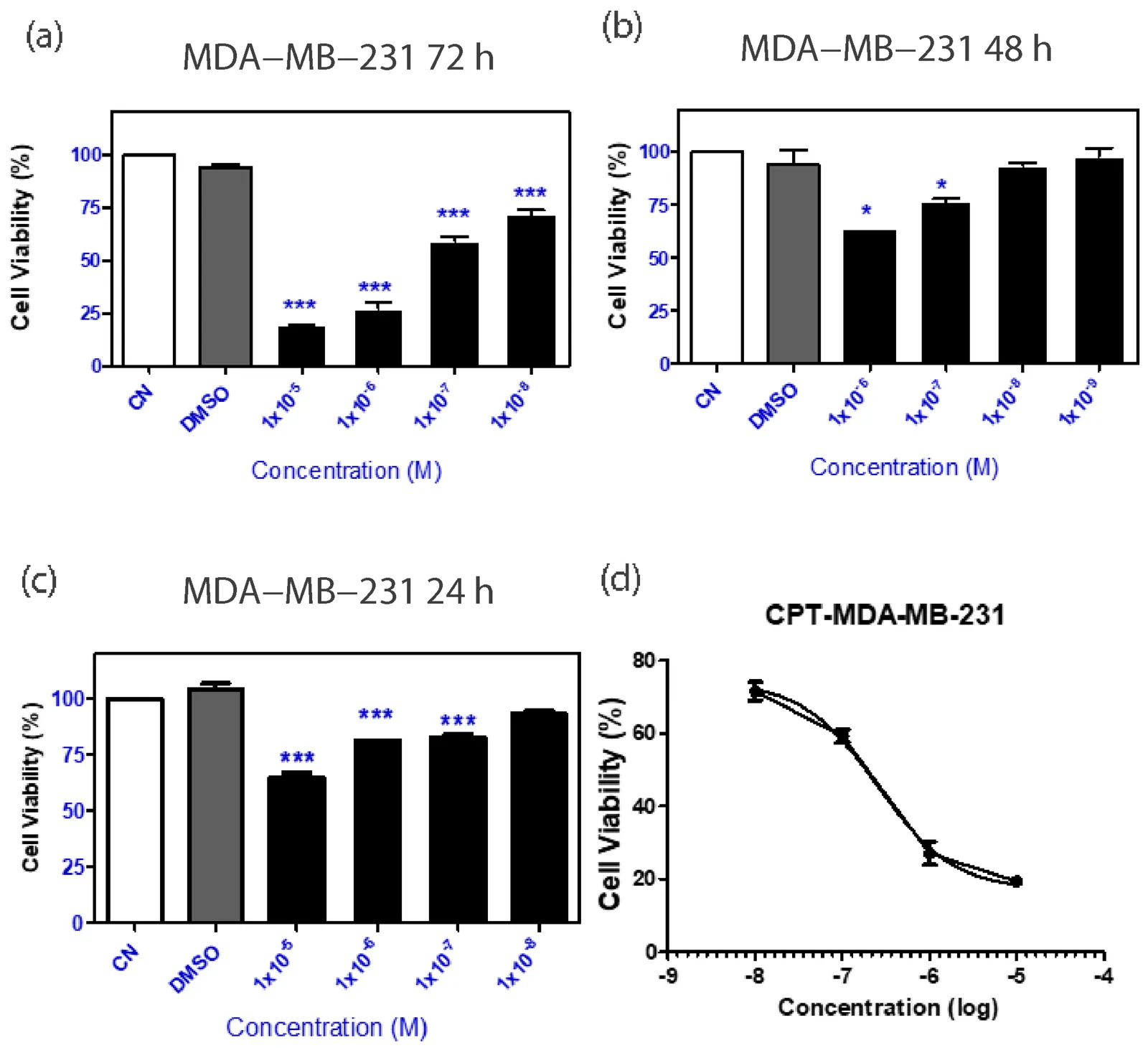
MTT-based cell viability of the MDA-MB-231 breast cancer cell line treated with camptothecin at different time points. Figure (**a**) shows results at 72 h post-treatment, figure (**b**) at 48 h, and figure (**c**) at 24 h. Figure (**d**) represents the dose–response inhibition curve. All assays were performed in triplicate, and statistical analysis employed ANOVA followed by Tukey’s post hoc test at a 95% confidence level (* *p* < 0.05) (*** *p* < 0.001).

Figure 8 presents the viability assay results at 24, 48, and 72 h for the MDA-MB-231 triple-negative breast cancer cell line following exposure to camptothecin at concentrations ranging from 1 × 10^−8^ to 1 × 10^−5^ M. Camptothecin was dissolved in DMSO as the vehicle, and cells were serum-starved for 12 h before treatment. The first two bars or panels denote the control groups: CN (untreated cells) and DMSO (cells treated with DMSO at the same concentration used in the experiments). The results indicate a clear concentration-dependent effect, with higher concentrations producing greater inhibition relative to controls. At the highest concentration tested, inhibition approached 80% of cell viability. The figure indicates results for the 48 h time point in the MDA-MB-231 line, showing a statistically significant difference only at 1 × 10^−7^ M and 1 × 10^−6^ M, with approximately 40–60% inhibition of cell proliferation. Figure 8d presents the dose–response inhibition curve used to determine the half-maximal inhibitory concentration (IC_50_), which was calculated to be 2.52 × 10^−7^ M.

#### 2.1.10. Cell Migration Assays in the MDA-MB-231 Cell Line

**Figure 9:**
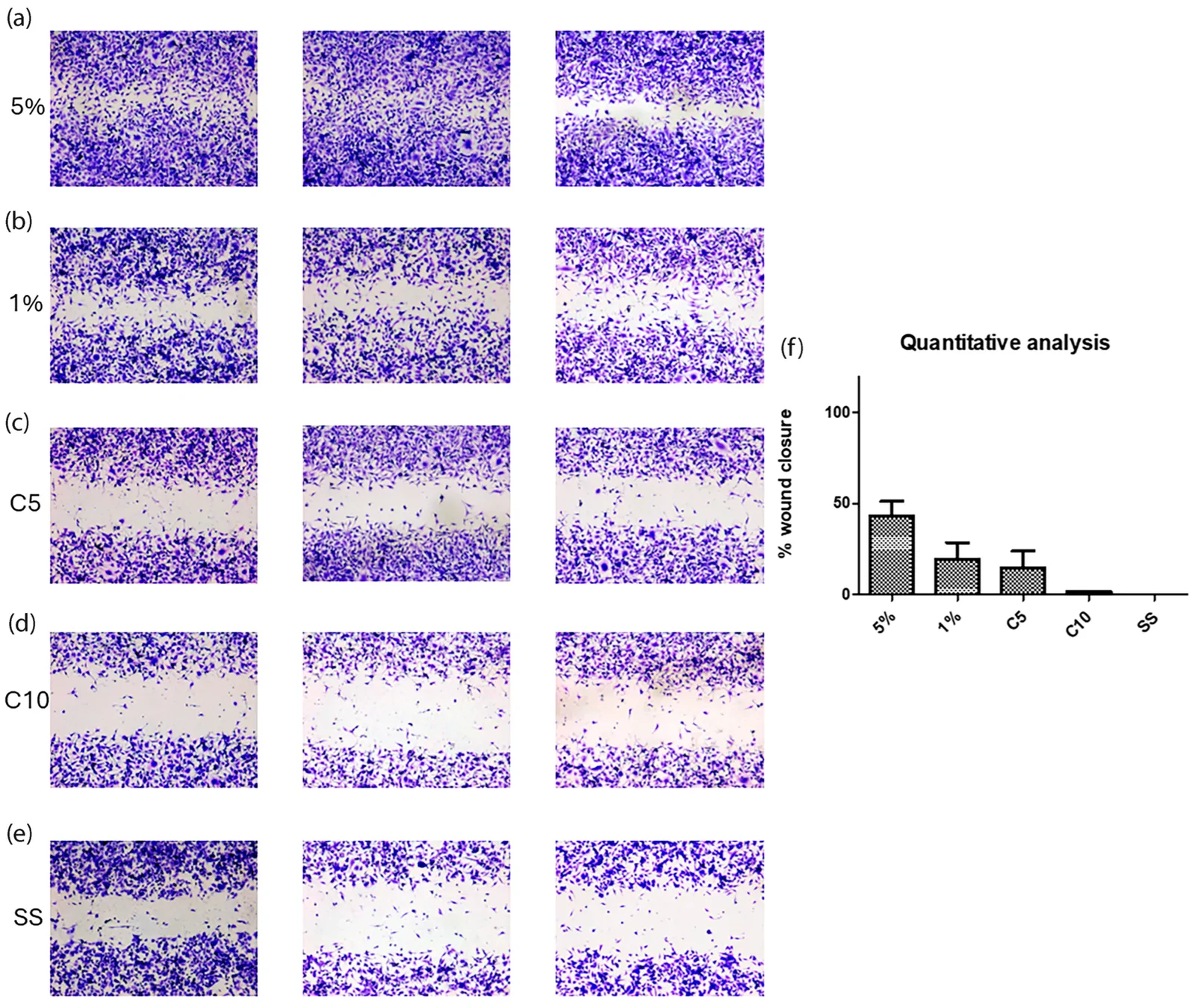
24 h wound closure trial with camptothecin. Figure (**a**) contains triplicate of the tests carried out with 5% serum. Figure (**b**) corresponds to 1% fetal bovine serum, figures (**c**,**d**) are the results of the administrations of camptothecin at two different concentrations and finally part (**e**) corresponds to the tests carried out in medium without fetal bovine serum. The quantitative analysis of wound closure is presented in figure (**f**).

Figure 9 presents the results of the wound-healing (migration) assay performed 24 h after treatment, following the protocol described in Methods. The assay was conducted in triplicate. Figure 9a shows the negative control condition with medium and 5% fetal serum; the cells exhibit normal growth, and the wound is approximately 90% closed after 24 h. Figure 9b depicts the low-serum control (1% serum), representing serum starvation; partial wound closure is still observed despite the reduced serum. Figure 9c represents camptothecin at 1 × 10^−7^ M, where no additional wound closure is observed relative to the control. Figure 9d shows camptothecin at 1 × 10^−6^ M; in triplicate, the wound remains open, indicating inhibited cell migration at this concentration. Figure 9e is the positive migration control (complete absence of fetal serum), in which cells are unable to migrate, and the wound remains open. As shown in Figure 9f, quantitative analysis of wound closure revealed that the 1 × 10^−6^ M concentration markedly inhibited wound closure, producing an effect comparable to that observed in the serum-free culture medium control (SS).

## 3. Discussion

In silico analyses suggest that camptothecin may interact with key receptors implicated in breast cancer, including EGFR, HER2, integrins, and estrogen receptor (ER). This finding implies a mechanism of action that complements camptothecin’s established inhibition of topoisomerase I, indicating a dual effect: (i) disruption of membrane receptor-mediated growth and adhesion signaling, and (ii) direct DNA damage via topoisomerase I inhibition. Such dual activity could enhance efficacy in certain tumor phenotypes, particularly where EGFR/HER2 and integrin signaling drive proliferation, migration, and therapeutic resistance. In vitro, treatment reduced viability and migration in the MDA-MB-231 and MCF-7 cell lines. Considering that MCF-7 is ER/PR-positive and MDA-MB-231 is a triple-negative line with higher migratory capacity, these results imply that camptothecin might be effective in breast cancer subtypes that are less responsive to hormonal or HER2-targeted therapies. This has implications for combination therapies and for tumors with heterogeneous receptor status. Moreover, the predicted interaction with EGFR/HER2/integrins raises the possibility of synergistic effects with existing treatments (e.g., ER/HER2 inhibitors, anti-EGFR therapies) or with membrane-targeted delivery approaches (e.g., nanomedicine, surface ligands). Pairing camptothecin with targeted delivery strategies could enhance tumor selectivity and mitigate systemic toxicity. The integration of molecular docking and affinity assessments with viability and migration assays strengthens the interpretation, with concordant decreases in proliferation and migration and predicted receptor affinity supporting a membrane-target-mediated component in addition to the canonical topoisomerase I mechanism. The observed antiproliferative effect in both cell lines appears to be, at least at the tested concentrations, independent of hormonal status or ER/HER2 expression. This breadth of activity is encouraging for potential indications beyond hormone receptor-positive disease, including receptor-negative tumors. The reduction in MDA-MB-231 migration observed in a wound-healing assay further suggests that camptothecin may impede migratory and possibly metastatic processes, a finding particularly relevant given the aggressiveness of this cell line as a model of metastasis. Finally, camptothecin and its derivatives are known to face toxicity and pharmacokinetic challenges; therefore, formulation strategies, targeted delivery, and conjugation approaches (e.g., nanoparticles, liposomes) should be explored to improve tumor selectivity and reduce adverse effects.

## 4. Materials and Methods

Breast cancer cell lines MDA-MB-231 and MCF-7 were purchased from the American Type Culture Collection (ATCC, Manassas, VA, USA) (HTB-26™) and (HTB-22™). The breast cancer cell line was grown in a monolayer.

### 4.1. Cell Cultures

MDA-MB-231 and MCF-7 breast cancer cells were cultured in DMEM medium (high glucose) supplemented with 5% fetal bovine serum (FBS) and 1% penicillin–streptomycin (*v*/*v*) dissolved in PBS for both cell lines. The cells were incubated at 37 °C with a humidified atmosphere at 5% CO_2_.

### 4.2. In Vitro Cytotoxicity Measurements

In vitro cytotoxicity was evaluated by means of the MTT colorimetric assay following 24, 48, and 72 h of treatment. Prior to cell exposure, the CPT was prepared in DMSO and sonicated for 15 min to facilitate complete dissolution and stored at 4 °C. Cells were seeded into 24-well culture plates at a density of (1 × 10^5^) cells/well and allowed to attach for 24 h. For the viability assessment, 125 μL of MTT solution was added to each well, and the plates were maintained at 37 °C for 3.5 h. The resulting culture medium was subsequently removed, and 300 μL of DMSO was added to dissolve the intracellular formazan crystals. Plates were agitated at 130 rpm for 15 min on an orbital shaker to promote homogeneous dissolution. Untreated cells maintained in DMEM containing 2.5% fetal bovine serum served as the control group. Absorbance was recorded at 590 nm using a Bio-Rad™ Model 680 microplate reader (Laboratories, Inc., Hercules, CA, USA). Cell viability was calculated relative to the untreated control based on the corresponding optical density measurements. All experimental conditions were analyzed in three independent replicates.

### 4.3. Cell Migration Assay

For the cell migration assays, only the MDA-MB-231 breast cancer cell line was used because of the observed inhibitory effect of camptothecin. Then, 30 mm culture dishes were prepared to promote cell growth and adhesion. Cells were plated in medium containing 5% FBS, and once 90% confluence was reached, a wound was created with a 200 µL micropipette tip. The medium was aspirated, and fresh medium containing varying concentrations of fetal serum was added to each experimental group. After 24 h, the assay was terminated; cells were fixed and stained with crystal violet to enhance contrast. Three subsequent washes were then performed to remove excess stain and residues. The serum-free culture medium group was used as the control for comparison of the percentage of wound closure.

### 4.4. Statistical Analysis

The data were presented as the mean of triplicates ± SD. The results were analyzed using a one-way ANOVA with Tukey’s test, and significance was set at *p* < 0.05.

### 4.5. Molecular Docking Set

The two-dimensional structures of all ligands were first constructed using BIOVIA Draw 2022 [25]. These molecular representations were subsequently converted into three-dimensional coordinates with Avogadro [26]. The resulting geometries were then subjected to energy minimization using Gaussian 09W [27] through the semi-empirical PM3 method [28], as implemented in GaussView 5.0. This optimization procedure was performed to obtain energetically favorable and geometrically refined ligand conformations, providing suitable three-dimensional structures for subsequent molecular docking calculations.

For the molecular docking, the protein structures of the two receptors, HER-2, EGFR, integrin, and estrogen receptor were obtained from the Protein Data Bank (PDB) (https://www.rcsb.org/) [29]. Specifically, the HER2 receptor was represented by PDB code: 3PP0 [30], while the EGFR receptor was assigned PDB code: 1M17 [31]. The integrin receptor was assigned by PDB code: 9IUJ, and the estrogen was represented by two different proteins by PDB code: 2IOG and 7KBS. It should be noted that two estrogen receptor structures were employed, designated RE2 for the 2IOG receptor and RE7 for the 7KBS receptor to prevent confusion. The primary distinction between the structures lies in their protein length: RE7 comprises 265 amino acids with four mutations, whereas RE2 comprises 246 amino acids with no mutations. The receptor structures were obtained from the corresponding PDB entries, which provided the three-dimensional coordinates required for the computational analysis. Prior to docking, the protein models were processed by eliminating nonessential ligands and crystallographic water molecules, followed by the addition of hydrogen atoms and appropriate atomic charges. Protein preparation was carried out using Discovery Studio Visualizer 2021 [32] and AutoDockTools 1.5.7 [33]. Missing amino acid residues were reconstructed and modeled with the CHARMM-GUI web server, following the procedure described in the PDB Reader and Manipulator module [34,35].

Molecular docking calculations were subsequently performed with AutoDockTools 1.5.7 using a blind-docking strategy. In this approach, the complete receptor surface was considered rather than restricting the search to a predefined binding pocket, allowing the identification of both known and potentially unexplored or allosteric ligand-binding regions. The Lamarckian genetic algorithm [36] was employed for the conformational search, using an initial population of 100 individuals and a maximum of 10,000,000 energy evaluations. Ligand molecules were allowed to undergo conformational changes during the simulations, whereas the receptor structures were maintained as rigid bodies.

For the HER2 receptor, a grid box of 126 × 126 × 126 Å was employed with a grid spacing of 0.494 Å and was centered on the receptor structure. The EGFR calculations used a 126 × 90 × 78 Å grid with a spacing of 0.87 Å, also centered on the corresponding protein. For the integrin receptor, the grid dimensions were 66 × 104 × 120 Å with a spacing of 1.00 Å. The estrogen receptor structure 2IOG was analyzed using a 126 × 126 × 126 Å grid with a spacing of 0.51 Å, whereas the 7KBS structure was evaluated using a 126 × 126 × 126 Å grid with a spacing of 0.47 Å. In all cases, the grid box was centered on the respective receptor. The Lamarckian genetic algorithm was used to explore alternative ligand orientations and conformations, and the resulting docking solutions were ranked according to their predicted binding free energy. The poses presenting the most favorable energy values were subsequently selected for interaction analysis.

The predicted ligand–receptor complexes were examined using AutoDockTools 1.5.7 and Discovery Studio Visualizer. These programs were used to characterize and visualize the principal noncovalent interactions established between the docked ligands and receptor residues, including hydrogen bonding and hydrophobic contacts. The resulting computational workflow, encompassing receptor preparation, ligand docking, pose selection, and interaction analysis, was used to characterize the potential molecular interactions of the evaluated compounds with the selected receptor targets.

### 4.6. Molecular Dynamics

The conformational stability of the protein–ligand complexes obtained from the docking calculations was subsequently investigated by molecular dynamics (MD) simulations using the GROMACS package [37]. The complexes displaying the most favorable docking energies were selected for further analysis, and the ligand and receptor coordinates were initially separated using BIOVIA Discovery Studio Visualizer. Ligand structures were converted to Sybyl Mol2 format with Avogadro to facilitate topology generation, after which the resulting files were adapted as required for use in GROMACS. Ligand parameters and topology information were generated through the CGenFF web platform. For the receptor components, topological files were prepared with the GROMACS pdb2gmx utility using the all-atom CHARMM36 force field [38].

The individual ligand and receptor coordinates were subsequently combined to generate the corresponding protein–ligand complexes. Each system was placed in a cubic simulation box and explicitly solvated using the TIP3P water model; SPC216 water was additionally considered for validation purposes. A minimum separation of 1.0 nm was maintained between the complex and the boundaries of the simulation box [39]. The systems were neutralized by the addition of either sodium or chloride counterions, as required, and the ionic strength was adjusted to 0.15 M.

Prior to the production simulations, each system underwent energy minimization for 5000 steps. Equilibration was then performed for 100 ps under both NVT and NPT ensembles, with the temperature maintained at 310 K and the pressure at 1.0 bar. Following equilibration, each complex was subjected to a 100 ns MD production run. During the simulations, several structural descriptors were monitored, including the root-mean-square deviation (RMSD), root-mean-square fluctuation (RMSF), radius of gyration (Rg), and solvent-accessible surface area (SASA), to evaluate the structural stability and dynamic behavior of the complexes.

The stability profiles obtained for the ligand-bound systems were compared with corresponding simulations of the unbound receptor structures. Approximately 5000 trajectory frames were collected from each simulation for subsequent structural and dynamic analyses.

## 5. Conclusions

The results suggest greater selectivity of camptothecin (CPT) for the MDA-MB-231 breast cancer cell line than for the MCF-7 line. This observation argues against a primary role for CPT binding to membrane receptors such as EGFR and HER2, although involvement of these receptors to a lesser extent cannot be excluded. Moreover, CPT may interact with additional membrane targets, including estrogen receptors or integrins. Molecular modeling indicates a higher predicted affinity of CPT for estrogen receptors and for HER2 relative to standard breast cancer inhibitors. Migration assays indicate that CPT can impede breast cancer cell motility. To evaluate its therapeutic potential more comprehensively, CPT should be tested in other model systems. If CPT can simultaneously limit unchecked cell proliferation and reduce migratory capacity that contributes to metastasis, it could potentially improve outcomes for patients with this disease.

## Figures and Tables

**Table 1:** Results obtained from the coupling energies and the affinity constant between the ligands and the HER2 and EGFR receptors.

Ligand	HER2		EGFR	
	Energy (kcal)	Kd	Energy (kcal)	Kd
Neratinib	−8.02	1.32 μM	−5.84	52.13 μM
Erlotinib	−6.42	19.65 μM	−4.63	400.8 μM
Camptothecin	−8.32	796.19 nM	−6.92	8.4 μM
TAK	−6.46	18.34 μM	−3.42	3.13 mM

**Table 2:** Results obtained from coupling energies and the affinity constant between the ligands and the ER and Integrin receptors.

Ligand	ER				Integrin	
	G2 Energy	Kd	G7 Energy	Kd	Energy	Kd
Neratinib	−7.72	2.2 μM	−9.15	197.35 nM	−5.86	50.29 μM
Erlotinib	−7.16	5.68 μM	−7.27	4.71 μM	−6.0	39.95 μM
Camptothecin	−7.78	1.86 μM	−7.6	2.67 μM	−6.57	15.28 μM
TAK	−6.26	25.77 μM	−8.47	613.78 nM	−4.34	658 μM

## Data Availability

Breast cancer cell lines MDA-MB-231 (accession number: CRM-HTB-26) link https://www.atcc.org/products/crm-htb-26 and MCF-7 (accessed on 30 April 2025) (accession number: HTB-22) link https://www.atcc.org/products/htb-22 (accessed on 20 April 2025) were purchased from the American Type Culture Collection (ATCC Manassas, VA, USA). All data reported is available upon request.
